# Multispectral intravital microscopy for simultaneous bright-field and fluorescence imaging of the microvasculature

**DOI:** 10.1186/s42649-021-00059-6

**Published:** 2021-07-24

**Authors:** Barry G. H. Janssen, Mohamadreza Najiminaini, Yan Min Zhang, Parsa Omidi, Jeffrey J. L. Carson

**Affiliations:** 1 Department of Medical Biophysics, Western University, London, ON N6A 5C1 Canada; 2 Kidney Clinical Research Unit (KCRU), London Health Sciences Centre, London, ON N6C 6B5 Canada; 3 Imaging Program, St.Joseph’s Health Care, Lawson Health Research Institute, London, ON N6A 4V2 Canada; 4 Department of Pathology, Western University, London, ON N6A 5C1 Canada; 5 Trauma Research Centre, Fourth Medical Center of the Chinese PLA General Hospital, Beijing, 100048 People’s Republic of China; 6 School of Biomedical Engineering, Western University, London, ON N6A 3K7 Canada; 7 Intensive Care Unit, Tianjin Nankai Hospital, Tianjin, 300100 People’s Republic of China

**Keywords:** Multispectral microscopy, Brightfield microscopy, Fluorescence microscopy, Intravital microscopy

## Abstract

Intravital video microscopy permits the observation of microcirculatory blood flow. This often requires fluorescent probes to visualize structures and dynamic processes that cannot be observed with conventional bright-field microscopy. Conventional light microscopes do not allow for simultaneous bright-field and fluorescent imaging. Moreover, in conventional microscopes, only one type of fluorescent label can be observed. This study introduces multispectral intravital video microscopy, which combines bright-field and fluorescence microscopy in a standard light microscope. The technique enables simultaneous real-time observation of fluorescently-labeled structures in relation to their direct physical surroundings. The advancement provides context for the orientation, movement, and function of labeled structures in the microcirculation.

**Supplementary Information:**

The online version contains supplementary material available at 10.1186/s42649-021-00059-6.

## Introduction

### The microcirculation and intravital microscopy

The microcirculation is an essential physiological, anatomical structure for organ and tissue function. It enables blood cells and plasma to be transported into tissue and metabolic waste products to be removed. Conventional bright-field intravital light microscopy (IVM) allows the visualization of the microvasculature in great detail and can be used to investigate its role in tissue function during various physiological and pathophysiological conditions (James and Tanke 2012). IVM has been used to investigate leukocyte-vessel wall interactions, essential for effective immunological response (Pinho et al. 2011). Furthermore, using an intravital microscope equipped with a dual-filter camera system, it has been possible to investigate both changes in tissue perfusion and in-vivo RBC oxygen content (Ellsworth et al. 1987). Other researchers have used IVM for the investigation of the behavior of platelets and thrombus formation (Jenne et al. 2011; Celi et al. 2003), vascular growth (Sckell and Leunig 2016), and tumor formation and metastasis (Beerling et al. 2011).

### Intravital light microscopy and its limitations

In intravital microscopy, conventional light sources such as halogen, xenon or mercury lamps are used to illuminate the tissue. Bright-field illumination involves trans-illumination of the surgically exposed transparent tissue, while epi-illumination can be used to visualize the microvasculature in thicker tissues (James and Tanke 2012). IVM often involves the observation of fast moving red blood cell (RBC) through microvessels, it is usually necessary to enhance the RBC visibility as much as possible. Since hemoglobin both in its deoxygenated and oxygenated form, have high optical densities between 400 and 450 nm (Zijlstra et al. 1991; Nitzan et al. 2014), optical filters selective for this spectral range are deployed to enhance image contrast and RBC visibility. Multispectral observations in living tissue have been performed using a dual camera system using 2 separate digital cameras (Japee et al. 2005a; Japee et al. 2005b). Although this allowed to investigate both tissue perfusion and RBC oxygen levels in vivo, the use of separate cameras can create challenges in alignment and image acquisition synchronization (Ellsworth et al. 1987).

Visualizing other less visible components in the blood stream generally requires the use of specific fluorescent labeling to augment optical visibility. Fluorescence microscopy requires a specific bandpass filter illuminate the tissue with light containing the fluorochrome’s excitation wavelengths, while a second bandpass filter ensures that only the emitted fluorecencent light can be visualized. Fluorescent labeling of moving cells in the microvasculature has been successfully used to investigate the in-vivo behavior of leukocytes and platelets (Janssen et al. 1994; Al-Khazraji et al. 2012; Oude Egbrink et al. 2002). Moreover, although different fluorochromes can be used concurrently, it is usually not possible to visualize different fluorochromes simultaneously during microscopy, since each fluororescent label requires a different filter set to visualize the emitted fluorescent light (James and Tanke 2012; Coling and Kachar 2001).

Up to now, conventional intravital light microcopy has not been optimized for simultaneous bright-field and fluorescence microscopy. Mainly due to the limitations of the emission filter set, which only allows fluorescent light to pass, impeding simultaneous intravital brightfield and fluorescence microscopy. In standard laboratory IVM setups it is therefore, not possible to capture images using both modalities concurrently but rather, bright-field and fluorescence images need to be recorded separately. Since microvascular blood flow velocities can range up to approximately 10,000 μm/sec (Al-Khazraji et al. 2012; Tangelder et al. 1986), observing any moving component in the microvascular blood flow becomes extremely challenging and the manual switch between the different modalities would result in a direct misregistration of brightfield and fluorescent images, thereby obstructing any effective temporal resolution of the captured microscopic images.

### Objective and approach

This work aimed to evaluate if a multispectral imaging technique could perform simultaneous fluorescence/bright-field imaging during an IVM observation in living tissue. Our approach was to extend an IVM setup with a multispectral (MS) system that allowed for simultaneous bright-field and fluorescence observation through a standard, inverted light microscope. We tested the new multispectral intravital microscopy (MSIVM) setup by capturing fluorescence images simultaneously at different emission wavelengths and by combining fluorescence with conventional bright-field images at video frame rates. The system was tested on a phantom model before in-vivo testing on the microvasculature of a rat. With the new MSIVM setup, we acquired images that captured the microvascular flow dynamics simultaneously with fluorescently labeled structures that would typically not be visible with conventional IVM.

## Materials and methods

### Animal preparation

All experimental animal work described in this study was performed in accordance with the legal guidelines and regulations set by the Canadian Council of Animal Care (CCAC) (https://www.ccac.ca), and approved by the Animal Care and Use Committee (ACUC) of Western University, London, Ontario, Canada. All animal work in the study was carried out in compliance with the ARRIVE guidelines (https://arriveguidelines.org) (Percie du Sert et al. 2020).

Male Sprague-Dawley rats, purchased from Charles River (Wilmington, MA, USA), were housed under standard conditions, i.e., water and food ad libitum, 12/12 light-dark cycle at room temperature. The animals were allowed to acclimatize for at least 72 h after arrival at the animal facility before entering the experimental procedures.

All animals (200–250 g body weight) were fully anesthetized with isoflurane (induction 4%, maintenance 2–2.5%) and permitted to breathe freely during the experiment. Maintenance anesthesia was tapered to minimize its effects on blood pressure. Body temperature was maintained at 37 °C using a rectal probe and an infrared lamp connected to an automated temperature monitor (TCAT-2 Temperature Controller, Physitemp Instruments Clifton NJ). A 24G intravenous catheter was placed in the tail vein to facilitate direct IV delivery of suspended microbeads; fluid resuscitation was used with heparinized (1 U/ml) 0.9% sodium chloride (600 μl/100 g body weight/hr) to ensure patency of the catheter during the experiment.

### Surgery

Microvascular observations were performed on the Extensor Digitorus Longus (EDL) muscle, as previously described (Tyml et al. 1991). Briefly, the EDL muscle was exposed and separated from the surrounding tissue, after which the distal tendon was tied and detached from the bone. After exposing the muscle tissue, the animal was transferred to an inverted optical microscope, and the exposed muscle was gently placed on a thin glass coverslip. The exposed muscle tissue was regularly super-fused with physiological saline, covered with saran wrap, and a glass coverslip to isolate the muscle from the surrounding environment and prevent desiccation of the tissue. To minimize tissue movement, a suture at the tendon was used to hold the muscle in place and keep the muscle at the approximately in-situ length without impeding microvascular blood flow.

### Overview of multispectral multicamera system

The MSIVM system was designed to accommodate the classical principles used for intravital microscopy (James and Tanke 2012) (Spectral Devices, London (ON), Canada; https://www.spectraldevices.com). While in conventional microscopes, filters are either placed in front of the camera or, in the case of fluorescence microscopy, in optical cubes within the microscope body, the MSIVM system allowed for filters to be placed within the optical path of each camera. This design feature allowed for various filter possibilities, which were suited to the observational circumstances. Since the four individual optical light paths in the MSIVM system were designed to act independently, specific filters in one light path could be used without interfering with the image acquisition in other optical arms. Therefore, we could tailor the optical characteristics for each camera within the MSIVM system. Although this could be easily achieved for image acquisition in the two bright-field optical arms, specific filter selection was required for simultaneous fluorescent and bright-field imaging. This approach contrasts with conventional fluorescence microscopy, where excitation and emission light needed to be well separated by filters with non-overlapping optical ranges to generate usable fluorescent images (Coling and Kachar 2001). In the MSIVM system, we used selective illumination of the tissue using light in an optical range that only contained the fluorochromes’ excitation wavelengths (i.e., wavelength range from 400 to 550 nm). This approach had several advantages since it allowed for clear bright-field visualization of the RBCs in the microvasculature and efficient excitation of several fluorochromes of interest. Moreover, since a high-intensity xenon-light source was used for the intravital observations in this study, optical filters were used that excluded UV and IR light, which could potentially damage the tissue during the observation. Combining this illumination strategy with specific band pass and long pass filters enabled the simultaneous and separate visualization of two fluorescent images in the first two optical arms and bright-field images in the third and fourth optical arms of the MSIVM.

### Multispectral multicamera setup

**Fig. 1:**
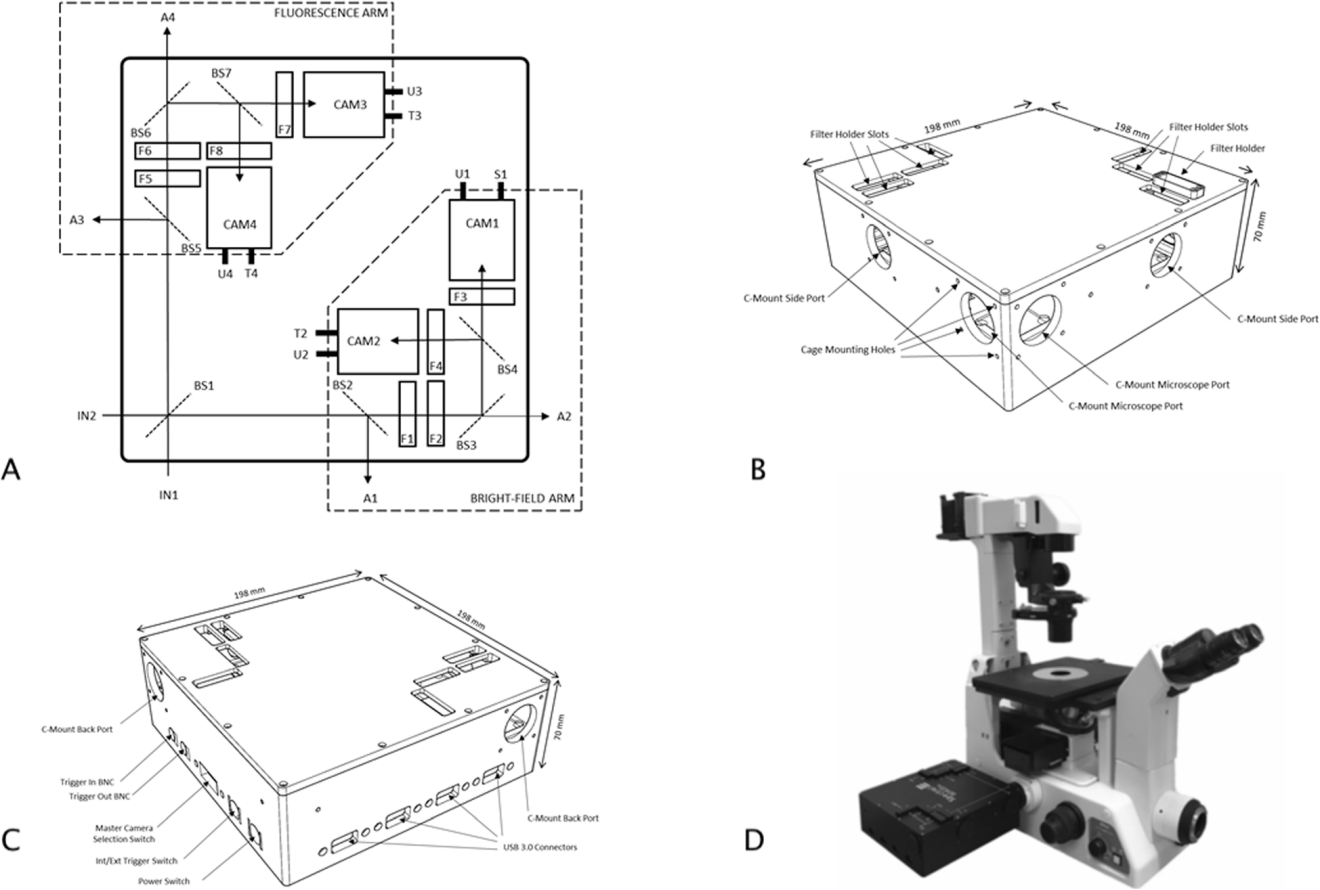
Schematic diagram of the MSMC-23-1-A showing the bright-field and fluorescence optical paths. (**A**): The instrument includes up to seven beam splitters (BS1 - BS7), four accessory ports (A1 - A4), eight filter holders (F1 - F8), four cameras (CAM 1 - CAM 4), and two input ports (IN1, IN2). The USB 3.0 port on each camera is connected to a 4-port USB 3.0 interface card in the PC workstation. Internally, the strobe output (S1) from CAM1 is connected to the trigger inputs (T2, T3, and T4) on the other 3 cameras (CAM2, CAM3, and CAM4). (**B**): Front perspective of the MSMC-23-1-A showing C-mount ports, filter holder slots, with one slot populated with a filter holder. (**C**): Rear perspective of the MSMC-23-1-A, showing C-mount backports, USB port connections, trigger and power connectors, and switches. (**D**): Photograph of the MSIVM system with MSMC-23-1-A attached to the side port of an inverted microscope

Figure 1 shows the MSIVM system (MSMC-23-1-A, Spectral Devices Inc., London, Canada) consisting of four 2.3MP CMOS cameras (Omron Sentech, Japan, Model STC-MBS231U3V) and several beam splitters to create four separate optical arms. Figure 1A shows that one beam splitter (BS1) received incoming light from the microscope and divided the optical path into two arms. One arm was used for bright-field imaging and the other for fluorescence imaging. Each optical arm was folded with either a mirror or beam splitter to reduce the overall system size. After each folding mirror/beam splitter, the light passed through another beam splitter, thereby splitting the light in each arm into two directions, which was ultimately received by two cameras. The system could pick off light from each optical arm using accessory beam splitters, but these were not installed for the experiments in this study. At four positions along each optical arm, there were holders for 25.4 mm optical elements. In total, the system provided four independent but co-registered imaging channels of the light exiting the side port of the microscope. This setup allowed us to combine two basic illumination modalities, i.e., bright-field and fluorescent microscopy. For this investigation, only one bright-field channel was used in combination with 2 separate fluorescent channels. Perspective drawings of the MSMC-23-1-A are shown for the front of the unit (Fig. 1B) and the rear (Fig. 1C). A typical experimental arrangement of the MSIVM system on an inverted microscope is shown in Fig. 1D. Since fluorescent light usually has a low intensity, we used a 90/10 beam splitter (BS1 in Fig. 1A) to ensure that the two cameras used for fluorescent observation (CAM3 and CAM4 in Fig. 1A) received 90% of the emanated light, while the remaining 10% was used for the bright-field observation.

### Bright field intravital microscopy

Observations in the tissue were made using an inverted microscope (Nikon Eclipse Ti, Nikon Instruments, Melville, New York, USA) with a stage adapted for intravital microscopy. The EDL tissue was transilluminated using a 100 W xenon light source (PTI LPS 220, Horiba Scientific, Piscataway NJ, USA) combined with an optical light guide (Thorlabs, Newton, NJ, USA). The tissue was trans-illuminated with light in the range of 400–550 nm, while an additional filter (450 nm/20 nm band-pass filter; 450BP20, Omega Optical, Brattleboro, VT, USA) was placed in the light path directly in front of the bright-field camera. This approach enhanced the visualization of red blood cells (RBC), facilitated fluorescence microscopy, and prevented any tissue damage related to UV and IR light emanating from the xenon light source. Intravital images of the EDL microcirculation (see Fig. 1C) were acquired using the bright-field CMOS camera units of the MSIVM system and were stored on a computer for later off-line analysis.

### Fluorescence intravital microscopy

Fluorescence microscopy tests were performed using two different types of fluorescent microbeads; (FluoroSpheres: diameter 1.0 μm; excitation wavelength 540 nm, emission wavelength 560 nm; 1*10^10^ microbeads /ml; Molecular Probes, Inc., Eugene, OR, USA) and 1.0 μm fluorescent microbeads (TransFluoroSpheres: diameter 1.0 μm; excitation wavelength 488 nm, emission wavelength 645 nm; 3.6*10^10^ microbeads /ml; Molecular Probes, Inc., Eugene, OR, USA).

### In vitro visualization of microbeads

A quantity of 1*10^8^ fluorescent microbeads was suspended in saline (total end volume of 1 ml) and vortexed before use. Of each microbead suspension, 10 μl was taken separately or mixed on a glass slide and covered with a glass coverslip for microscopic inspection. Fluorescent microscopic images of the microbeads were acquired using the fluorescence CMOS monochrome cameras of the MSIVM system in combination with a band-pass filter (570/25 nm; 570IL25 Comar Optics, Linton, UK) and long-pass filter (> 600 nm; FEL0600, Thorlabs, Newton, NJ, US).

### In vivo visualization of the microbeads

A quantity of 4*10^9^ beads was mixed in a total end volume of 0.5 ml to achieve a final concentration of 8*10^9^ microbeads/ml. The suspension was vortexed before injection into the infusion line connected to the 24G intravenous (IV) catheter. The microbeads were slowly infused at a rate of 750 μl/100 g body weight/hr. for 10 min. Intravital fluorescence images of the microbeads traveling through the microvasculature of the EDL microcirculation were acquired using the fluorescence CMOS monochrome camera units of the MSIVM system in combination with a band-pass filter (565/24 nm; MF565–24, Thorlabs, Newton, NJ, US) and long-pass filter (> 600 nm; FEL600, Thorlabs, Newton, NJ, US).

### Off-line analysis

All acquired images were subsequently stored on a computer for off-line analysis. We developed an application in MATLAB app designer (Matlab; The Mathworks Inc., Natick (MA) USA) to perform co-registration among three separate camera images. The application was also capable of reducing artifacts from tissue movement during the experiment. For co-registration, we utilized the control point registration software in MATLAB Image Processing Toolbox™. This software allowed us to select geometrical features (landmarks) in an image from the reference camera and apply them to the images from the other cameras. A 2-dimensional transformation matrix for each camera was generated to co-register all camera images based on the selected landmarks. A similar method was used to select fixed landmarks among all frames from one camera and co-register all frames based on the chosen landmarks to remove tissue movement. This processing resulted in a transformation matrix for each frame applied to the corresponding frames from the other cameras. Finally, to show a single multispectral video for all cameras, synchronized frames from all cameras were overlaid using color coding to visualize the interactions between microbeads and RBCs (see “Availability of data and materials” section). ImageJ (NIH, USA; https://imagej.nih.gov/ij/) was used for basic image editing (cropping and resizing) and visualization.

## Results

### Simultaneous bright-field and fluorescent imaging

**Fig. 2:**
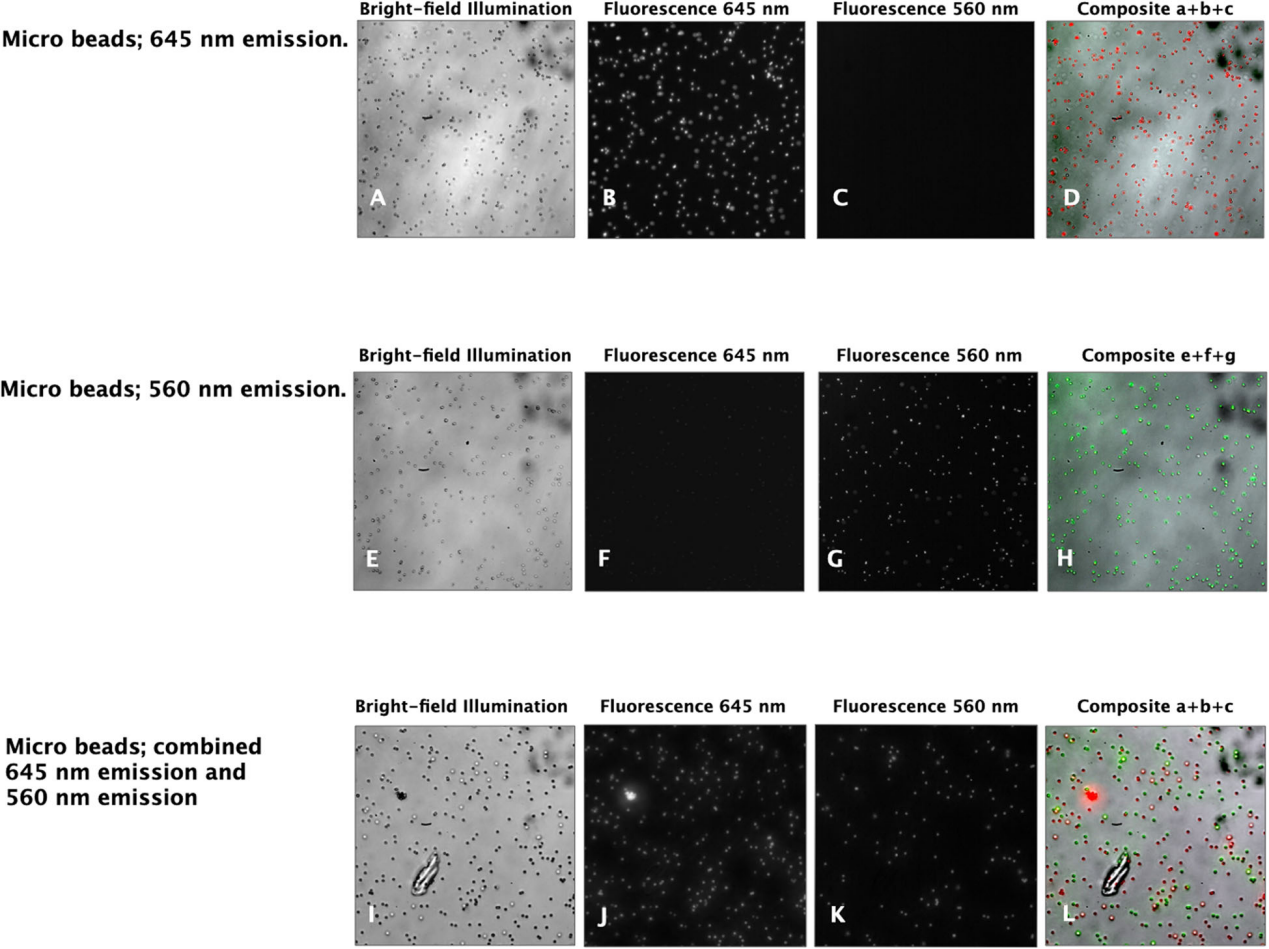
Fluorescent microbeads on a glass slide. Images (20X objective; 1000 × 1000 pixels; 93 μm × 93 μm) of microbeads (diameter: 1 μm) on a glass slide, observed with optical channels of the MSIVM system. Images of microbeads with emission at 645 nm are shown for the bight-field channel (400-550 nm, **A**), the > 600 nm fluorescence channel (**B**), the 570/25 nm fluorescence channel (**C**), and the composite channel (**D**). Images of microbeads with emission at 560 nm are shown for the bright-field channel (400-550 nm, **E**), the > 600 nm fluorescence channel (**F**), the 570/25 nm fluorescence channel (**G**), and the composite channel (**H**). Simultaneous vizualization of a mixture of two types of fluorescent microbeads (emission at 560 nm and 645 nm) on a glass slide shown for the bright-field channel (**I**), the > 600 nm fluorescence channel (**J**), the 570/25 nm fluorescencechannel (**K**), and the composite channel (**L**). Microbeads: excitation 488 nm/emission 645 nm and excitation 540 nm/emission 560 nm. Illumination wavelength range: 400–550 nm. Filters: band-pass filter (570/25 nm) and long-pass filter (600 nm). Please note that since all images are captured on monochrome cameras in the MSIVM system, no color images can be generated directly. We used image processing to add color for visualization purposes only

The MSIVM system allowed the visualization of bright-field and fluorescent images simultaneously. Images taken from the two different microbead types are shown in Fig. 2A-D and Fig. 2E-H showing images from the different optical channels, i.e., bright-field and the 645 nm and 560 nm fluorescent channels. While Fig. 2A and Fig. 2E exhibit a bright-field image of the microbeads, Fig. 2B, F, and C and g show the respective fluorescent channels. The images were combined in a composite image of the bright-field and corresponding 645 nm (red) and 560 nm (green) fluorescent images, as shown in Fig. 2D and Fig. 2H, respectively. Figure 2I-L show images of a mixture of the two different bead types and the composite image, which demonstrated that the MSIVM system could simultaneously and separately image the different bright-field and separate fluorescent optical channels.

### In-vivo visualization of microbeads

**Fig. 3:**
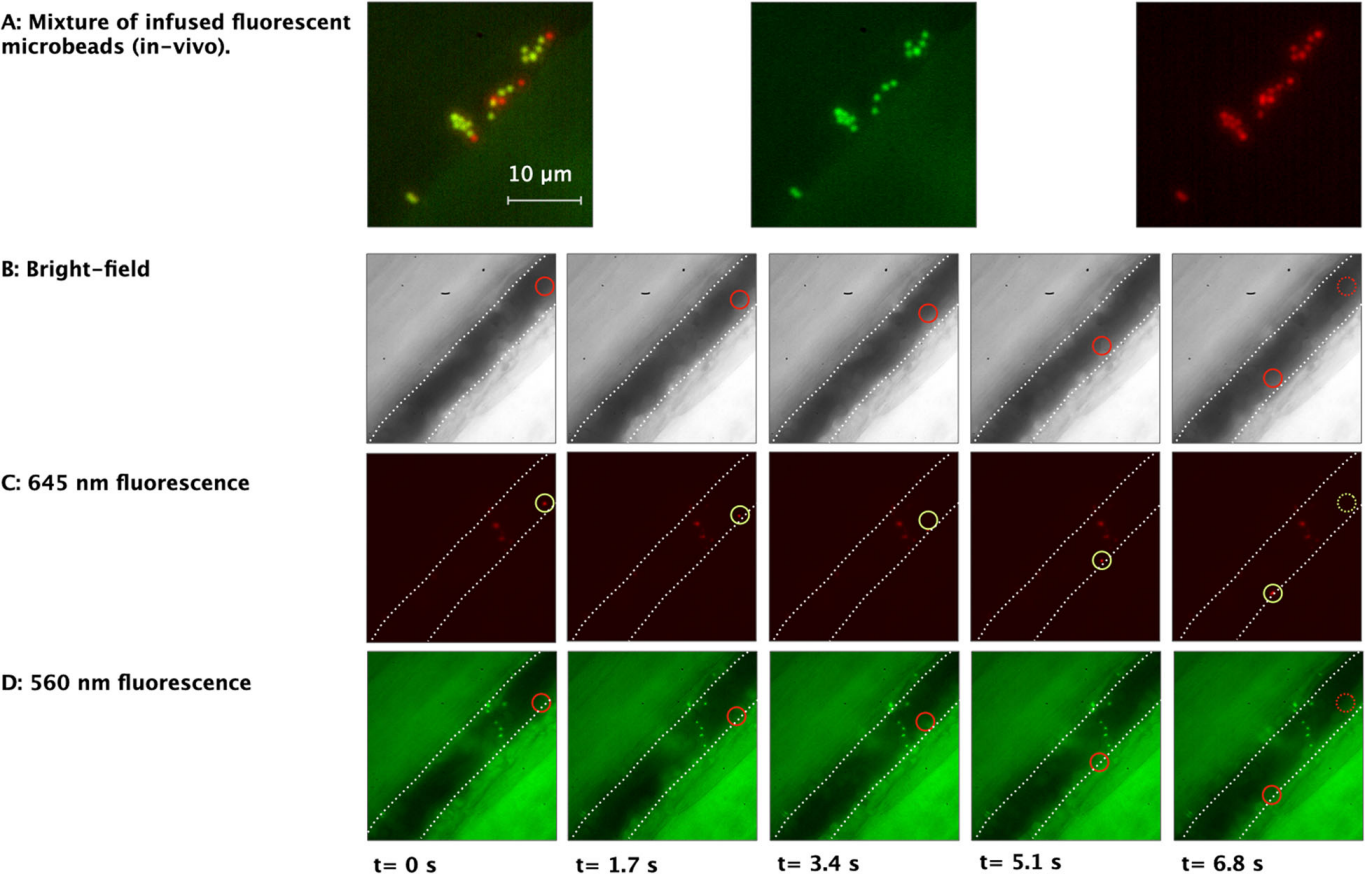
Mixture of infused fluorescent microbeads in-vivo. (**A**): *left image:* Composite images of adherent infused fluorescent 1 μm microbeads, *middle* *image*: green fluorescent (560 nm) microbeads, and *right image*: red fluorescent microbeads (> 600 nm). Notice that the green/yellow microbeads in the composite image represent green beads visible (green channel) and, due to an optical overlap of the 560 nm emission spectrum are also visible in the red channel. In the composite picture, these appear as yellow-green, clearly distinguishable from the red fluorescent microbeads. The orange microbeads result from a positional overlap between 2 different beads (in red and green channel repsectively). (**B**), (**C**), and (**D**): Show a sequence of intravital microscopy images of a microbead moving along the vascular endothelium. Images in (**B**) and (**D**), represent the image sequences displaying the location (red circles) of the fluorescent bead visible only in image sequence (**C**) (yellow circles). Bright-field illumination wavelength range: 400–550 nm (**B**). Filters: band-pass filter: 565/24 nm (**C**) and long-pass filter: 600 nm (**D**). Microbeads: excitation 488 nm / emission 645 nm (**C**) and excitation 540 nm / emission 560 nm (**D**). Exposure: 40 ms; frame rate: 16.7 fps. Please note that since all images are captured on monochrome cameras in the MSIVM system, no color images can be generated directly. We used image processing to add color for visualization purposes only. White dotted lines indicate the approximate position of the vascular wall

Figure 3A: left image shows a cluster of injected fluorescent microbeads adhered to the vascular wall in a vessel in the EDL muscle after infusion into the bloodstream. Since a 600 nm long-pass filter was used for these intravital observations, there was a slight optical overlap with the emission spectrum of 560 nm microbeads, whereas some optical leakage of emitted fluorescent light in the > 600 nm channel was observed. Consequently, the 560 nm microbeads were visible in the image associated with the > 600 nm channel. Although optical leakage between channels was difficult to avoid due to the fluorochromes’ emission peak width, our results show that it was possible to distinguish differently labeled structures within tissue. The 560 nm (green/yellow) microbeads show up in the composite picture as yellow/orange colored and, therefore, are distinguishable from the 645 nm microbeads, visible as deep red (Fig. 3A: middle image and right image, respectively).

### Dynamic intravital bright-field and fluorescence imaging

Simultaneous imaging in bright-field and fluorescence channels allowed the visualization of structures and dynamic processes that would not be visible with bright-field microscopy alone. For example, Fig. 3B,C and D show a postcapillary venule (diameter ~ 22 μm) at select time points from three simultaneously acquired image sequences, i.e., image sequences with bright-field illumination (Fig. 3B) and fluorescence microscopy (Fig. 3C and D, respectively). While a moving fluorescent microbead can be observed in the image sequence of Fig. 3C (yellow circle), it is not visible in the concurrent bright-field (Fig. 3B) or fluorescent (Fig. 3D) image sequences. Moreover, since these images are acquired at a frame rate of 16.7 frames per second, it is possible to determine the velocity with which this microbead moves along the vessel wall.

**Fig. 4:**
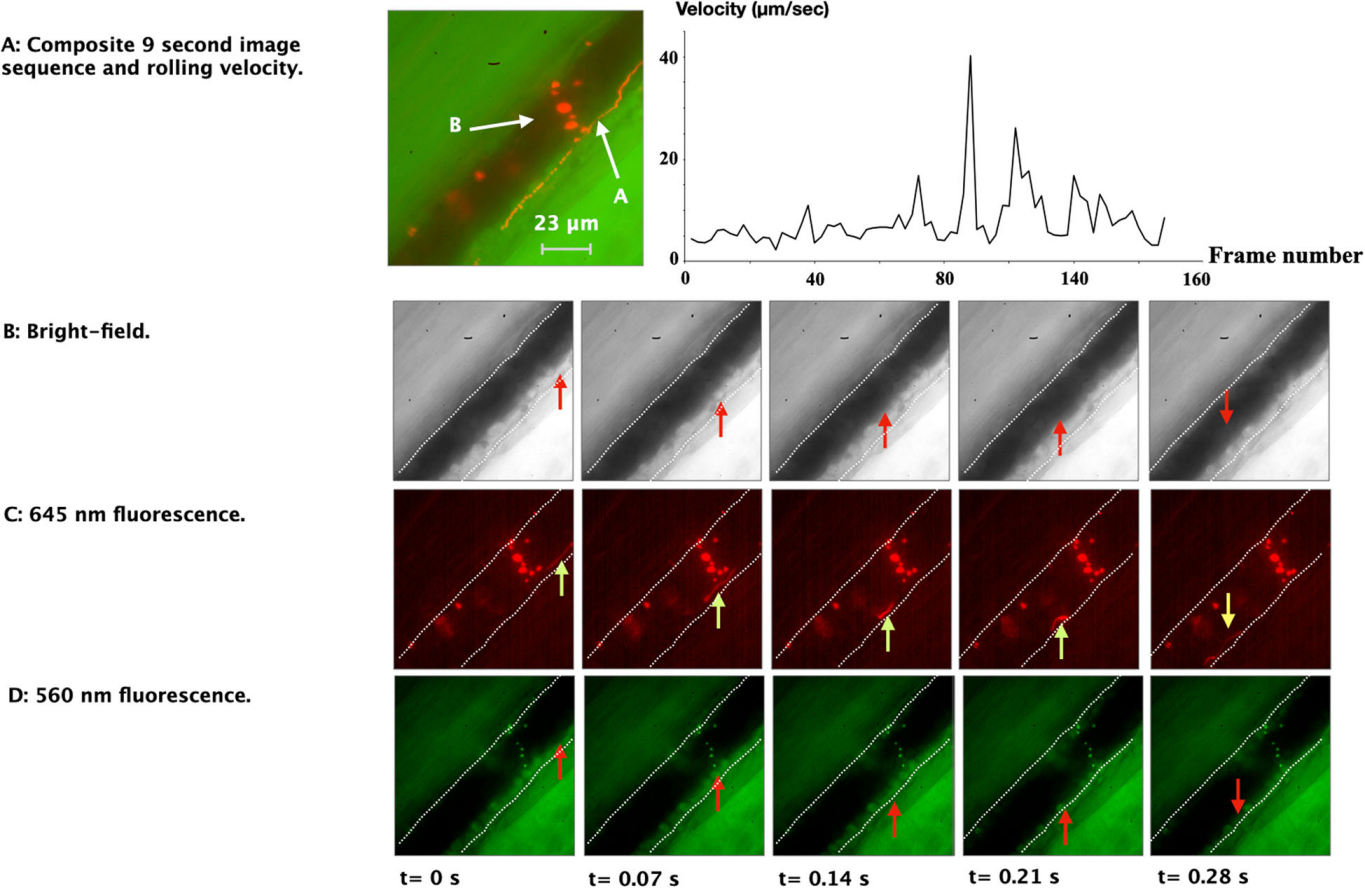
Composite picture of a 9 s image sequence of 150 images (red and green channel). (**A**): *left image:* The composite image shows the path (white arrow A) of a moving microbead observed in the fluorescent > 600 nm optical channel. A group of non-moving, adherent microbeads is also visible (white arrow B); *right image:* Velocity of microbead A in left image of panel (**A**). The distance of the microbead was estimated in each pair of successive images. Velocity was computed by determining the traveled distance in the interframe interval. (**B**), (**C**) and (**D**): Intravital microscopy image sequence of a microbead (streak) moving in the bloodstream’s centerline. With an image exposure time of 40 ms, this free-flowing fluorescent microbead’s average velocity was 468 μm/s (range: 270–710 μm/s). Arrows in image sequences (**B** and **D**), displaying the location of the fluorescent bead only visible in image sequence (**C**) (yellow arrow). Bright-field illumination wavelength range: 400–550 nm (**B**). Filters: band-pass filter: 565/24 nm (**C**) and long-pass filter: 600 nm (**D**). Microbeads: excitation 488 nm / emission 645 nm and excitation 540 nm/ emission 560 nm. Exposure: 40 ms; frame rate: 16.7 fps. Please note that since all images are captured on monochrome cameras in the MSIVM system, no color images can be generated directly. We used image processing to add color for visualization purposes only. White dotted lines indicate the approximate position of the vascular wall

To examine the complete 9-s long image sequence, a composite image of 150 images was generated (Fig. 4A: left image). Analysis of the frames revealed that the mean microbead velocity was approximately 7.9 μm/s, ranging from 2.3 to 40.1 μm/s (Fig. 4A: right image), which is considerably slower than the blood flow often found in these types of vessels (Lipowsky 2005). Furthermore, since blood flow in the microcirculation generally involves a parabolic velocity profile across the vessel lumen, the result likely represents a slow-moving microbead flowing in the outer slow-flowing fluid layer bloodstream close to the endothelial wall (Tangelder et al. 1986; Koutsiaris 2009).

A microbead traveling in the fast-flowing center of the bloodstream is shown in Fig. 4B, C and D. The figure shows three simultaneously acquired image sequences taken using intravital bright-field (Fig. 4B) and fluorescence microscopy (Fig. 4C and D). A visible streak, generated by a free-flowing microbead, can be observed in the > 600 nm channel (Fig. 4C), which is not visible in the 560 nm channel (Fig. 4D). Since a 40 ms exposure time was used for each image, the free-flowing fluorescent microbead’s velocity was determined by measuring the fluorescent streak’s length (Al-Khazraji et al. 2012). We found that the observed microbead had an average speed of 468 μm/s (range: 270–710 μm/s).

### Tracking a rolling leukocyte in the microvasculature

**Fig. 5:**
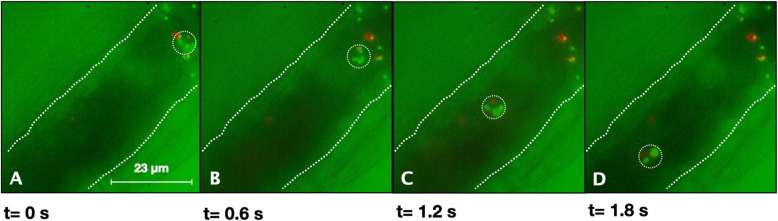
Composite intravital microscopy image sequence of a rolling leukocyte (red and green channel). Image sequence showing a sequence of still images (**A-D**) taken from a video sequence (see: Supplemental Video 1), revealing a rolling leukocyte with several adherent microbeads, emitting fluorescence at 600 nm (red) and 560 nm (green). With an image exposure of 40 ms, the free-flowing fluorescent microbead’s velocity ranged from 12 to 36 μm/s. Microbeads: excitation 488 nm / emission 645 nm and excitation 540 nm / emission 560 nm. Illumination wavelength range: 400–550 nm. Filters: band-pass filter (565/24 nm) and long-pass filter (600 nm). Exposure: 40 ms; frame rate: 16.7 fps. Please note that since all images are captured on monochrome cameras in the MSIVM system, no color images can be generated directly. We used image processing to add color for visualization purposes only. White dotted lines indicate the approximate position of the vascular wall

The intravital bright-field images revealed several rolling leukocytes that moved slowly along the vascular wall. Leukocyte rolling is the initial adhesive interaction between leukocytes and the vascular endothelium and facilitates the adhesion and extravasation in response to an inflammatory reaction. Leukocyte rolling is often observed in tissue that is surgically exposed for intravital microscopic observation (Alon and Feigelson 2002). Figure 5 shows a composite image of bright-field and fluorescence images at different time points. The image sequence reveals a rolling leukocyte with several adherent 1 μm microbeads emitting fluorescence at 600 nm (red) and 560 nm (green). Note that the leukocyte carries both types of fluorescent microbeads while moving slowly through the vessel along the vascular wall (see: Video 1 Supplemental data). With a framerate of 16.7 fps (inter-frame interval: 60 ms), the velocity of the rolling leukocyte was within a range from 12 to 36 μm/s.

## Discussion and conclusions

Our results show that MSIVM can successfully combine both bright-field and fluorescence microscopy into a single platform. The MSIVM system enabled simultaneous imaging with two different optical modalities at frame rates capable of visualizing the dynamic changes found in the microvasculature. Furthermore, the MSIVM system, comprised of four independent optical arms, allowed the use of multiple fluorescent probes. The strategic placement of separate optical filters allowed each optical arm to capture the different excitation and emission wavelengths independently and image them simultaneously.

Both in-vitro and in-vivo experiments showed that MSIVM could separately distinguish two types of 1 μm fluorescent microbeads in different optical channels, while in the bright field optical channel alone, no distinction could be made between these two types of microbeads. Additionally, we found that even in the case of an optical overlap between fluorescent emission spectra, it was still possible to observe the two microbead populations separately.

Our investigations showed that the MSIVM system allowed visualization of the dynamics of moving fluorescently labeled microbeads, either free-flowing in the center of the bloodstream, moving close to the vascular wall (Fig. 4), or bound to the cell surface of a leukocyte traveling through a micro-vessel (Fig. 5). The MSIVM system provided direct visual reference of the labeled structures within the surrounding tissue’s layout and the microcirculation. Separate visualization of differently labeled structures during MSIVM enabled independent tracking of these structures as they moved through the microvasculature. In our study, the structures were either free-flowing microbeads in the bloodstream that interacted with the vascular endothelium or moving leukocytes. The MSIVM system allowed separate visualization of these dynamic processes in the microvasculature, which is usually not possible with conventional bright-field light microscopy.

### Advancement of MSIVM over IVM

Both brightfield and fluorescence IVM have been used in a wide range of biological and biomedical research fields to study cell behavior directly in the surroundings of the living tissue often employing both brightfield and fluorescent imaging modalities (James and Tanke 2012; Choo et al. 2020). IVM has been used to investigate the in-vivo behavior of leukocytes in the blood stream and their adhesive interaction with the vascular endothelium (Tangelder et al. 1986; Al-Khazraji et al. 2012; Ley et al. 2008), thrombi formation (Furie and Furie 2005), as well as the dymamics of cell migration into tissues (Pai et al. 2013; Li et al. 2012; Beerling et al. 2016). IVM has been used to investigate the blood flow and and functionality of the microcirculation (Tangelder et al. 1986; Al-Khazraji et al. 2012; Tozer et al. 2005), as well as provided a better understanding of the functional microvascular hemodynamics and transport of oxygen (Ellsworth et al. 1987) or targeted therapeutic delivery of active particles (van de Ven et al. 2012).

To enhance the observation of cells and tissue structures, fluorescent labels are used to improve visibility for microscopic observation. In conventional fluorescent light microscopy, this requires the use of a filter cube in the light path to selectively visualize the fluorescence at the expense of blocking all other light from the specimen. Consequently, capture of brightfield images requires manual removal of the filter cube from the lightpath. If the sample does not contain any moving structures (e.g., fixated tissue slices or stationary cells), removal of the filter cube to visualize bright-field and fluorescence images is a well-accepted practice. However, when observing rapid dynamic processes in living tissue (e.g., the blood flow in the microcirculation), a manual swap between filter sets is higly impractical and ineffective due to the high temporal variability of the image.

This limitation of conventional IVM is circumvented by the MSIVM system, where the optical channel of the microscope is split into multiple separate optical channels. This approach is further advanced by the synchronized timing of the image acquisition on all the optical channels. Our investigations shows that when multi-channel synchronized IVM observation was combined with user-selected optical filters in each optical path, simultaneous bright-field and fluorescence microscopy on living specimens was possible and the simultaneous visualisation of the dynamic processes in the tissue was feasible.

### MSIVM and other imaging approaches

The setup of the MSIVM system also allows its used in combination with other imaging methodologies for the multispectral microscopic visualisation of in tissue. As an alternative to conventional fluorescence microscopy, both spinning disk confocal microscopy (SDCM) and two-photon microscopy (TPM) can be used for an effective visualisation of fluorescently labeled structures in living tissue (Choo et al. 2020). In both, the specimen is illuminated with focussed laser light using 2 spinning Nipkov disks to respectively point-illuminate the labeled specimen and subsequently guide the emanated fluorescent light through a pin hole to form a real image, which can be directly viewed through a microscope, or captured on a digital camera (Shin-Ichiro et al. 2002; Shimozawa et al. 2013).

Since these disks are spinning at high speed (up to 5000 rpm), scanning of tissue sections occurs very fast and frame rates up to a 1000 frames/sec can be reached. Moreover, the very short point illumination ensures that any phototoxic effects of the laser light are limited (Nakano 2002; Stehbens et al. 2012; Cox 2002).

In both SDCM and TPM focussed laser light is used to create fluorescent images generated in a thin tissue layer, with minimal interference from signals from other tissue areas. In SDCM, the focussed laser light only has a limited penetration depth and therefore, can only be used for imaging of thin superficial tissue slices, however, in TPM the NIR laser allows for a deeper penetration into the tissue. While a single low energy NIR photon cannot effectively excite the fluororescent label, the high concentration of focussed photons, will facilitate near simultaneous collisions and excitation of the fluochrome. Furthermore, the use of the low energy NIR photons would also reduce the development of any phototoxic effects in the tissue (Pai et al. 2013; Li et al. 2012; Shimozawa et al. 2013; Helmchen and Denk 2005).

Like conventional fluorescence microscopy, both techniques described above use a bandpass excitation filter in the final optical light path to form the final fluorescent real image that can be visualized. As such, the optical signal generated in the tisse can likelwise be divided over multiple optical arms with different sets of optical filters. As such, it should be possible to integrate the MSIVM system directly with SDCM or TPM imaging, and enable the use of multiple fluorescent probes and to simultaneously create an image from different fluorescent signals.

### Limitations of this study

A drawback of the current MSIVM system was that light coming from the microscope was shared by four separate optical arms, which reduced the light intensity at each camera compared to a single optical path. Although the use of a 90/10 beam splitter ensures that most of any emanated fluorescent light will be captured by the cameras, visualization of any weak fluorescent signal may still prove to be challenging. However, dichroic beam splitters could replace the existing beam splitters, which would result in much higher efficiency in light transmission within each selected band. Furthermore, the images in this study were not enhanced with noise reduction and signal enhancement image processing algorithms, which could have revealed weak fluorescent signals in the captured images.

Another consideration for the use of the MSIVM system relates to data processing. Since each camera could acquire digital images at video frame rates up 40 fps, the resulting image data volumes exceeded 100 Gb per experiment. Clearly, at these data rates, the manageability of the image dataset becomes very challenging. Automated image processing tools will be essential for handling these large data sets in the future. Future developments will also involve improving image processing techniques and software to efficiently analyze large data volumes associated with MSIVM experiments (Mahmoud et al. 2020).

### Applications that could benefit from MSIVM and future directions

Therefore, we anticipate that the MSIVM platform can be successfully integrated in other microscopic imaging platforms that will allow for a further in-depth investigation of the dynamics of the physiological mechanisms in tissue. For example, since the microcirculation provides a conduit to transport plasma with numerous different bioactive substances and suspended cells into the tissue’s smallest blood vessels. Numerous IVM studies have examined the microvasculature’s functionality in various tissues and different pathological circumstances using either bright-field or fluorescence microscopy (Tuma et al. 2008). The microvasculature represents a unique physical environment, comprising both slow (e.g., vascular endothelium and vessel geometry) and fast dynamic structures and processes (e.g., immune responses, blood coagulation, and the investigation of plasma flow), which are difficult to visualize. Therefore, fluorescent probes are often required for the necessary visualization. It indicates that the ability to combine both bright-field and fluorescence IVM with the MSIVM system is especially valuable since it allows us to investigate these dynamic microvascular changes in direct relation to their physical surroundings.

## Conclusions

The MSIVM platform allows simultaneous visualization of the movement of RBC with conventional bright-field illumination while at the same time enabling the use of fluorescent probes to image the less visible elements in the blood flow (e.g., blood platelets and plasma). It allows for a detailed study of microvascular blood flow under different pathophysiological conditions, e.g., during a severe inflammatory response. This response, generally associated with a hyperinflammatory cytokine storm, often induces significant blood flow disturbances (microvascular dysfunction), resulting in organ failure and permanent tissue damage (De Backer et al. 2014; Zaim et al. 2020). Moreover, microvascular dysfunction is also associated with diabetes (Stehouwer 2018), obstructive sleep apnea (Karaman Koç et al. 2019), heart failure (Nelson et al. 2018), autoimmune conditions (Bordy et al. 2018), stroke, and Alzheimer’s disease (Rost et al. 2018; Jiang et al. 2018), indicating that it is found in a wide range of different pathologies. As such, the MSIVM platform can be used to visualize disturbances of the microvascular blood flow in great detail in a wide range of pathological conditions.

## Supplementary Information

**Additional file 1 **: **Video 1**.

## Data Availability

All data generated and analysed during this study are included in this published article and its Supplementary information files. The image processing software for co-registration and removal of any tissue movement (Co-registration and overlay) can be downloaded from the following link: https://www.mathworks.com/matlabcentral/fileexchange/73259-co-registration-and-overlay. Video 1 Combined composite movies of microbeads and a rolling leukocyte in the EDL muscle microcirculation of the rat (Supplemental Video 1). *Left panel*: Composite video of images of the bright-field, red and green optical channel. Right panel: Composite video of images of the red and green optical channel only. Movie sequence is created using the co-registration Matlab software​, which can be downloaded from the Mathworks link in the “Availability of data and materials” section. *Right panel:* Composite video of images of the red and green optical channel only; movie sequence created using ImageJ (https://imagej.nih.gov/ij/).

## Abbreviations

EDL: Extensor Digitorus Longus
IV: Intravenous
IVM: Intravital video microscopy
MS: Multi-spectral
MSIVM: Multispectral intravital video microscopy
RBC: Red blood cell
SDCM: Spinning disk confocal microscopy
TPM: Two-photon microscopy
